# Experimental Stand for Very-Low-Velocity Gas Flow Generation

**DOI:** 10.3390/s23031569

**Published:** 2023-02-01

**Authors:** Tymoteusz Piga, Waldemar Wodziak, Jacek Sobczyk, Andrzej Rachalski

**Affiliations:** Strata Mechanics Research Institute, Polish Academy of Sciences, 30-059 Krakow, Poland

**Keywords:** very-low-velocity gas flow, calibration, Mariotte’s bottle, laminar pipe flow

## Abstract

This article presents an experimental stand for the generation of gas flows with very low velocities. The constant volume air flow was generated in the measurement area with the use of a Mariotte bottle and a water piston (water column in a tank). The air-flow velocity was determined from the change in the height of the water piston with time, which was measured via the difference between the pressure above the water surface and the pressure at the bottom of the water column. The tests were carried out in the mean velocity range of 0.35–66 cm/s. The five different velocities of air flowing out of the measurement pipe, measured with the particle image velocimetry method were compared with the results from the water piston method. The differences did not exceed 7%.

## 1. Introduction

Measurements of very low air-flow velocities require dedicated measuring instruments. During their design and prototyping, a standard of very low air-flow velocity is necessary. They also require regular calibration to function properly.

The generation of a very slow, stationary air flow in a wind tunnel using conventional technical solutions such as fans is a very difficult task. Characteristic problems associated with this type of solution are the occurrence of air-flow velocity pulsations, a nonuniform velocity distribution in the gas stream cross-section, and difficulties with the low velocity standard. One possible way to create very slow flow in the test chamber of an open-circuit wind tunnel is to use a suction-side manifold to which a combination of several Venturi nozzles are connected in parallel. This solution was applied in [1] and it allows for obtaining a velocity range from 4.5 to 140 cm/s in the measuring chamber.

Among calibration methods that do not require stationary air flow, there is a method that uses vortices shed from a cylinder [2,3,4]. The tested device is placed behind the cylinder, and the relationship between the Strouhal and Reynolds numbers is used to determine the flow velocity. There are also methods for calibrating measuring instruments by moving the sensors at a known velocity in stagnant air. Typical solutions include placing the calibrated hot-wire anemometer probe on a support: moving in a straight line [5,6,7], or moving in a rotating or pendulous motion [8,9,10,11]. The use of moving objects for calibration requires air stagnation around the calibrated probe and a reduction in vibration with the use of mechanical devices that support the probe.

One of the easiest methods of creating a slow flow is to use a long tube in which the flow is laminar. Andrews et al. [12] used a 2 m long pipe to calibrate a hot-wire anemometer at low velocities. Air was sucked from the pipe with a known volume flow. It was assumed that the flow in the pipe was laminar and fully developed, and that the maximal velocity along the axis of the pipe was twice as high as the mean velocity. Calibration was performed in a mean velocity range from 5 to 250 cm/s.

A calibration method using a water piston (tank) and a copper tube in which the flow was laminar was described by Yue and Malmstrom [13]. Water flowing into a vessel filled with air forced its outflow. This air flowed into a measuring pipe in which a hot-wire anemometer was mounted. The obtained results on the stand with a water piston were compared with the results obtained from a certified test stand. In the velocity range from 16.7 to 218 cm/s, the difference between them was 3.5%, and the greatest deviation was 4 cm/s. A tube with laminar flow was also used by Özahi et al. [14] to calibrate a hot-wire anemometer. The velocity range was from 2.9 to 179 cm/s. The flow was forced using a screw compressor, a buffer tank, and a pipe with a length of 146 *D_x_*, where *D_x_* is the pipe diameter. The results obtained from the pipe were compared with the results obtained by the spinning disk method. The maximal difference between the velocity values obtained using the two methods was 7%.

The aim of this study was to test the measurement method presented by Yue and Malmstrom [13]. Mass flow rate was measured by Yue and Malmstrom [13] “with balance weighing and watch”. This method is independent of the geometry of the piston. However, such a measurement stand is prone to ambient pressure fluctuations, so the measurement of average mass flow rate is insufficient. In this study, we propose the use of differential manometers to obtain the current flow rate. This method was verified by comparing the velocity profile measured with the PIV method at the pipe outlet with the theoretical profile determined on the basis of manometer readings.

## 2. Materials and Methods

**Figure 1:**
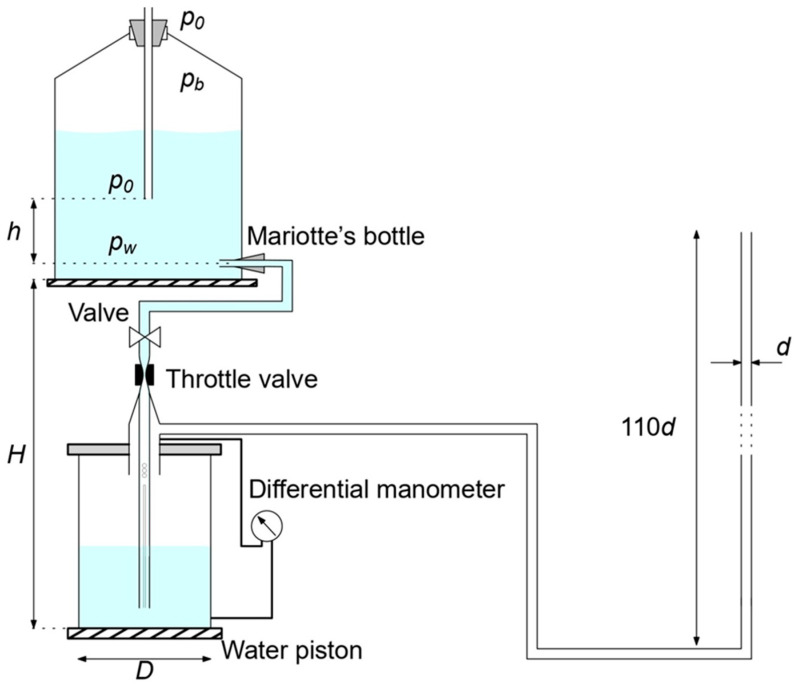
Measurement stand: *p*_0_—atmospheric pressure, *p_b_*—pressure above the water surface, *p_w_*—pressure at the bottom of the piston, *h*—distance between Mariotte’s bottle outflow level and the end of air supply pipe, *H*—distance between bottom of water piston and bottom of Mariotte’s bottle, *D*—diameter of water piston, *d*—diameter of outlet pipe.

The measuring stand using the water piston consisted of three basic elements: a Mariotte bottle, a water piston, and a measuring pipe (Figure 1).

The Mariotte bottle guarantees that the piston is supplied with a constant volumetric flow of water by maintaining constant pressure *p*_0_ at the level of the end of the tube immersed in the water. Pressure *p_b_* compensates for the pressure of the liquid above this level.

The outflow from the bottle is driven by the pressure difference of the water column at the height of drain hole *p_w_* and atmospheric pressure *p*_0_. The water flowing into the second container displaces the air inside it (the water surface forms a piston) and pushes it into the measuring chamber. In order to prevent the pressure of the water column in the water piston from affecting the velocity of the water outflow from the Mariotte’s bottle, the water supply pipe located in the piston chamber was perforated along its entire length. As long as the water level in the water piston does not rise above the perforations, water from the Mariotte’s bottle can flow freely into the piston. In such a situation, the main force counteracting with the outflow of water from the Mariotte’s bottle is the gas pressure in the water piston. Air flow stabilized in this way could be used for research and design purposes.

Under quasisteady conditions, the volume flows of water and air were equal to each other and could be measured by means of flow meters, optical, capacitive, or resistive water surface height sensors, a scale that continuously measures the mass of water in the piston, or pressure gauges determining changes in the pressure (height) of the water column in the piston [15].

## 3. Results and Discussion

### 3.1. Water Column Pressure Measurement

On the basis of the above-mentioned methods of determining the volume air flow, this study describes the measurement of the pressure change in the water column in the piston. The movement velocity of the water surface could be monitored with the use of a differential manometer measuring the pressure differences between the bottom of the water piston and the air above the water surface. The stand for generating the flow consisted of a Mariotte bottle enabling the production of a volumetric flow of water constant over time, a water piston with diameter *D* = 20 cm, and a tube with diameter *d* = 1.2 cm and length 132 cm (Figure 1). The length of the tube, equal to 110 *d*, allowed for the assumption of a fully developed laminar velocity profile [16]. The measuring chamber was placed in an upright position, which minimized the effect of thermal convection on the flow. The outlet of the tube was placed in the measuring chamber, shielding the outgoing air stream from external conditions. Heights h and H were selected to maximize the difference between the water outflow pressure and the atmospheric pressure while maintaining the desired active capacity of the Mariotte bottle, and they were *h* = 14.5 cm and *H* = 85 cm. Pressure *p_w_* at the bottom of the bottle, exerted by water and air, can be described by the following formula:

(1) $$p_{w}=p_{0}+\rho \cdot g\cdot h,$$

where

*p*_0_ is atmospheric pressure (Pa),
*ρ* is the water density (kg/m^3^),
*g* is acceleration due to gravity (m/s^2^),
*h* is the distance between Mariotte’s bottle outflow level and the end of air supply pipe (cm).

The height of the water column in the piston was measured using a Keller PD-41x differential manometer with a measuring range from 0 to 3000 Pa. The readings were converted directly into mH_2_O. The water volume flow was adjusted using a throttle valve. After changing the setting of the throttle valve, it was necessary to wait some time for a new state of dynamic equilibrium to be established between the Mariotte bottle and the water piston. It was assumed that at equilibrium the continuity equation was satisfied:

(2) $$V_{1}D^{2}=V_{2}d^{2},$$

where

*V*_1_ is the average velocity of the water surface in the piston (m/s),
*V*_2_ is the average air velocity in the outlet pipe (m/s),
*D* is the diameter of water piston (cm),
*d* is the diameter of outlet pipe (cm).

The value of the water surface velocity was calculated from the manometer readings. An expression for the water column height as a function of time was estimated using linear regression. The slope of the fitted line directly indicated the velocity of the water column *V*_1_. The uncertainty of the measurement of the volumetric flow $Q=V_{1}\frac{\pi }{4}D^{2}$ could be described with the following propagation uncertainty formula:

(3) $$\delta Q=\sqrt{{\left(\frac{\delta Q}{\delta V_{1}}\delta V_{1}\right)}^{2}+{\left(\frac{\delta Q}{\delta D}\delta D\right)}^{2}}=\sqrt{{\left(\frac{\pi }{4}D^{2}\delta V_{1}\right)}^{2}+{\left(2V_{1}\frac{\pi }{4}D\delta D\right)}^{2}}\;,$$

where

*δV*_1_ is the standard deviation of the linear regression of the velocity *V*_1_ (m/s),
*δD* is the uncertainty of the diameter of the water piston measurement, equal to 2 mm.

The value of *δV*_1_ is interpreted as the precision of the measurement. The average velocity in the outlet tube was calculated from the formula:

(4) $$V_{2}=\frac{4Q}{\pi d^{2}}\;,$$

The uncertainty of its measurement was:

(5) $$\delta V_{2}=\sqrt{{\left(\frac{\delta V_{2}}{\delta Q}\delta Q\right)}^{2}+{\left(\frac{\delta V_{2}}{\delta d}\delta d\right)}^{2}}=\sqrt{{\left(\frac{4\delta Q}{\pi d^{2}}\right)}^{2}+{\left(-\frac{8Q}{\pi d^{3}}\delta d\right)}^{2}},$$

where the measurement uncertainty of tube diameter *δd* was 0.1 mm.

Two series of 6 measurements of the water inflow to the water piston were performed for 12 different settings of the throttle valve. The corresponding volume flows were from 0.40 to 5.34 cm^3^/s and from 6.10 to 74.65 cm^3^/s. The highest obtained relative uncertainty was 2.7% for the first measurement series and 2.3% for the second series. The results are summarized in Table 1.

**Figure 2:**
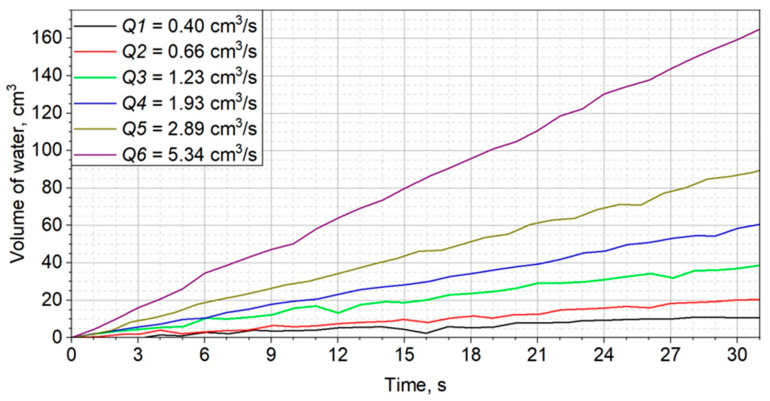
Manometer readings converted into water volume and volume flow in the range of 0.4–5.34 cm^3^/s.

**Figure 3:**
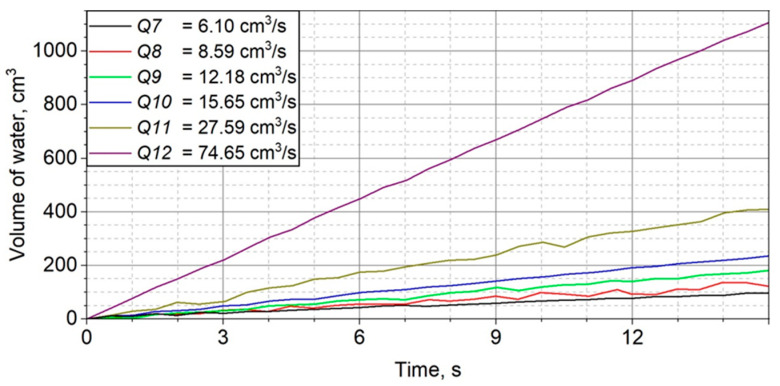
Manometer readings converted into water volume and volume flow in the range of 6.1–74.65 cm^3^/s.

The volumes of water flowing into the piston as a function of time are shown in Figure 2 and Figure 3.

The obtained values of the volumetric flow should correspond to the mean velocities in the tube, from 0.35 to 4.72 cm/s and from 5.39 to 66 cm/s, with an uncertainty of 2.5%. From the relationship of these values to the diameter of tube d, it was possible to determine the Reynolds numbers, which ranged from 0.3 to 525.

### 3.2. Gas Velocity Measurement at the Pipe Outlet

To verify the measurement method proposed by Yue and Malmstrom [13], the velocity of the gas outflow from the tube was tested using particle image velocimetry (PIV). The PIV method consists of introducing marker particles (seeding) into the examined area. Then, the seeding particles are illuminated twice with laser pulses of very short duration, forming a light sheet. A camera synchronized with the laser and positioned perpendicularly to the light sheet captures two frames. The particle pattern from the first image is sought in the second image. The single velocity vector is determined on the basis of the correlation of the two subareas (interrogation windows), from which the local mean particle displacement is obtained and related to the known time between the laser pulses. 

An Nd:YAG laser with a pulse energy of 200 mJ and a wavelength of 532 nm was used for the tests. A series of photographs was taken with a frequency of 5 Hz using an sCMOS camera with a maximal resolution of 2560 × 2160 pixels. The time between pulses depended on the tested velocity and ranged from 1.5 ms to 100 µs. The size of the interrogation windows was 32 × 32 pixels, and adaptive correlation was used to calculate the velocity fields. The actual dimensions of the examined area were 20 mm × 17 mm.

Measurements were conducted for five different throttle valve settings. DEHS (synthetic oil) seeding particles were applied directly to the piston and pipes before measurements. The laser sheet was placed in the pipe axis and perpendicular to camera optical axis. The measuring chamber was placed in a vertical position to minimize the influence of free convection on the air stream flowing out of the pipe. The obtained range of mean velocities, measured with the differential manometer, ranged from 1.10 to 31.27 cm/s. Each measurement was carried out under stable ambient temperature conditions. The air velocity distribution was analyzed as close to the pipe outlet as possible. Velocities measured with the differential manometer are denoted by a superscript M.

The theoretical radial velocity distribution in the pipe for laminar flow is as follows [16]:

(6) $$V\left(r\right)=V_{max}\left(1-{\left(\frac{r}{R}\right)}^{2}\right)\;,$$

where

*R* is the pipe radius (m),
*V_max_* is the maximum velocity, which is equal to 2·*V_avg_* (m/s),
*V_avg_* is the average velocity (m/s).

**Figure 4:**
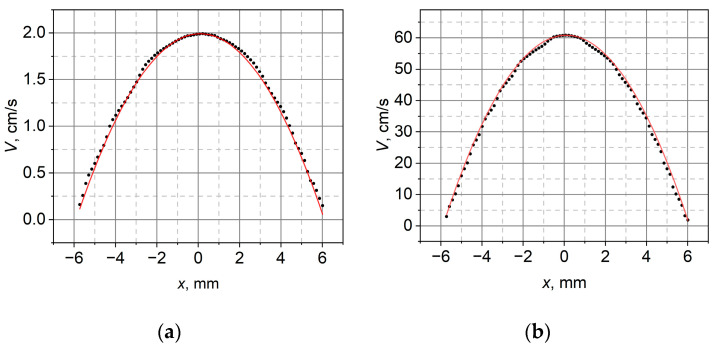
Velocity profile for setpoint: (**a**) $V_{avg}^{M}=1.1\;\mathrm{cm}/s$ and (**b**) $V_{avg}^{M}=31.27\;\mathrm{cm}/s$.

The velocity profiles recorded with the PIV method were very similar to the distribution given by (6). Figure 4 shows the obtained profiles for the experimental velocity values (dotted curves) and the adjusted theoretical relationships (solid red line).

Table 2 summarizes the results of gas velocity measurements in the pipe outlet performed using the two methods. To calculate the mean velocity on the basis of PIV measurements (denoted by a superscript P), a range of points with a length of 12.05 mm was taken, and due to axial symmetry, the weights of the measurement points were taken as π|x|dx for dx = 0.145 mm. This range corresponds to the diameter of the outlet opening. 

The differences between the mean velocities obtained by the two methods ranged from 4% to 7%. This corresponds well with the assumed uncertainty of the velocity determined with the use of a manometer. The differences between the maximal velocities are between 3% and 10%. The relatively large discrepancy in the case of the lowest velocity may have been caused by the very low kinetic energy of the gas flowing out into the measuring space, which rendered it susceptible to the influence of the environment.

## 4. Conclusions

An experimental stand for measuring very low velocities of flowing air consisting of a Mariotte bottle, a water piston, and a measuring pipe was presented in this article. The air-flow velocity in the pipe, calculated from the change in the height of the water column in the water piston, was verified using the PIV method. In the range of the obtained mean velocities from 1.1 to 32 cm/s, the discrepancy of the two methods ranged from 3% to 10%, with larger discrepancies at lower velocities. Except for the lowest velocity, the results of the measurements performed with the two methods were consistent in terms of their uncertainty. The discrepancies between the measurement of the gas velocity at the outlet of the pipe and the measurement of the water volume flow in the piston were primarily related to the precision of the determination and stability of the geometric dimensions of the water piston along its entire height, and the diameter of the outlet pipe. The experimental stand, in its present state or scaled up, can be used for the low velocity and flow rate calibration of anemometers and flowmeters. 

## Figures and Tables

**Table 1:** Summary of volumetric flow measurement results with uncertainties.

No.	*Q* (cm^3^/s)	*δQ* (cm^3^/s)	$\frac{\partial Q}{Q}\cdot 100\%$(%)
1	0.4	0.01	2.7
2	0.66	0.01	1.8
3	1.23	0.03	2.1
4	1.93	0.02	1
5	2.89	0.03	1
6	5.34	0.05	1
7	6.1	0.08	1.3
8	8.59	0.18	2.3
9	12.18	0.15	1.2
10	15.65	0.17	1.1
11	27.59	0.33	1.2
12	74.65	0.76	1

**Table 2:** Summary of calculated manometer indications of $V_{avg}^{M}$  with the measured average velocity $V_{avg}^{P}$  and measured maximal velocity $V_{max}^{P}$.

**No.**	$V_{avg}^{M}\left(\frac{cm}{s}\right)$	$V_{avg}^{P}\left(\frac{cm}{s}\right)$	$\frac{V_{avg}^{M}-V_{avg}^{P}}{V_{avg}^{M}}\cdot 100\%$	$2\cdot V_{avg}^{M}\left(\frac{cm}{s}\right)$	$V_{max}^{P}\left(\frac{cm}{s}\right)$	$\frac{2\cdot V_{avg}^{M}-V_{max}^{P}}{2\cdot V_{avg}^{M}}\cdot 100\%$
1	1.10	1.03	6.57	2.21	1.99	9.86
2	2.27	2.19	3.58	4.54	4.40	3.19
3	5.14	4.90	4.81	10.29	9.90	3.73
4	9.73	9.28	4.63	19.45	18.84	3.12
5	31.27	29.20	6.62	62.54	60.82	2.76

## Data Availability

Not applicable.
